# Estimation of Aboveground Biomass in Agroforestry Systems over Three Climatic Regions in West Africa Using Sentinel-1, Sentinel-2, ALOS, and GEDI Data

**DOI:** 10.3390/s23010349

**Published:** 2022-12-29

**Authors:** Dan Kanmegne Tamga, Hooman Latifi, Tobias Ullmann, Roland Baumhauer, Jules Bayala, Michael Thiel

**Affiliations:** 1 Department of Remote Sensing, Institute for Geography and Geology, Julius-Maximilians University of Würzburg, Oswald-Külpe-Weg 86, 97074 Würzburg, Germany; 2 Department of Photogrammetry and Remote Sensing, Faculty of Geodesy and Geomatics Engineering, K. N. Toosi University of Technology, Teheran P.O. Box 15433-19967, Iran; 3 Department of Physical Geography, Institute for Geography and Geology, Julius-Maximilians University of Würzburg, Am Hubland, 97074 Würzburg, Germany; 4 Centre for International Forestry Research (CIFOR)—World Agroforestry (ICRAF), Sahel, 06, Ouagadougou 06 BP 9478, Burkina Faso

**Keywords:** biomass modelling, agroforestry systems, remote sensing, West Africa, map uncertainty assessment

## Abstract

Agroforestry systems (AFS) offer viable solutions for climate change because of the aboveground biomass (AGB) that is maintained by the tree component. Therefore, spatially explicit estimation of their AGB is crucial for reporting emission reduction efforts, which can be enabled using remote sensing (RS) data and methods. However, multiple factors including the spatial distributions within the AFS, their structure, their composition, and their variable extents hinder an accurate RS-assisted estimation of the AGB across AFS. The aim of this study is to (i) evaluate the potential of spaceborne optical, SAR and LiDAR data for AGB estimations in AFS and (ii) estimate the AGB of different AFS in various climatic regions. The study was carried out in three climatic regions covering Côte d’Ivoire and Burkina Faso. Two AGB reference data sources were assessed: (i) AGB estimations derived from field measurements using allometric equations and (ii) AGB predictions from the GEDI level 4A (L4A) product. Vegetation indices and texture parameters were generated from optical (Sentinel-2) and SAR data (Sentinel-1 and ALOS-2) respectively and were used as predictors. Machine learning regression models were trained and evaluated by means of the coefficient of determination (R^2^) and the RMSE. It was found that the prediction error was reduced by 31.2% after the stratification based on the climatic conditions. For the AGB prediction, the combination of random forest algorithm and Sentinel-1 and -2 data returned the best score. The GEDI L4A product was applicable only in the Guineo-Congolian region, but the prediction error was approx. nine times higher than the ground truth. Moreover, the AGB level varied across AFS including cocoa (7.51 ± 0.6 Mg ha^−1^) and rubber (7.33 ± 0.33 Mg ha^−1^) in the Guineo-Congolian region, cashew (13.78 ± 0.98 Mg ha^−1^) and mango (12.82 ± 0.65 Mg ha^−1^) in the Guinean region. The AFS farms in the Sudanian region showed the highest AGB level (6.59 to 82.11 Mg ha^−1^). AGB in an AFS was mainly determined by the diameter (R^2^ = 0.45), the height (R^2^ = 0.13) and the tree density (R^2^ = 0.10). Nevertheless, RS-based estimation of AGB remain challenging because of the spectral similarities between AFS. Therefore, spatial assessment of the prediction uncertainties should complement AGB maps in AFS.

## 1. Introduction

The aboveground biomass (AGB) of a terrestrial ecosystem represents the quantity of dry vegetation per unit area, generally presented in tons per ha (Mg ha^−1^). It corresponds to the amount of carbon taken from the atmosphere through photosynthesis and indicates the amount of carbon that will be released in the atmosphere if the ecosystem is disturbed through deforestation, wood extraction or wildfire [1]. The estimation of the AGB is crucial to evaluate and monitor changes in ecosystems for the assessment of their contribution to climate change [2]. Good practice guidance (GPG) was proposed by the International Panel on Climate Change (IPCC) to ensure accurate estimation of the AGB with relatively no over- nor underestimation, in which uncertainties are additionally quantified [3]. The GPG requires the methodologies used for AGB estimations to be transparent, documented and assessed for uncertainties [4]. Thus, estimations of AGB reported under the GPG could be acknowledged as effort to combat climate change in the framework of the Paris agreement and could be eligible to rewarding mechanism such as reducing the emissions related to deforestation and deforestation (REDD+).

The method for AGB estimation is based on the combination of field measurements and remote sensing (RS) analysis. A good practice is to use the appropriate allometric equation (suitable for the ecosystem and the region) to generate AGB estimations from field measurements [3]. Field measurements are crucial for AGB modelling since it is used as reference for developing and testing AGB models. However, it is spatially limited and costly. As a solution, spaceborne RS emerged as the most efficient tool for modelling and monitoring ecosystems globally, providing consistent data in near-real time at almost no cost. RS data derived from passive and active sensors have been successfully used in AGB modelling: the multispectral optical data from the Sentinel-2 mission was reported to provide good predictors for AGB estimation in different wooded ecosystems [5,6,7,8]. Likewise, sentinel-1 data, providing synthetic aperture radar (SAR) C-band information, returned a higher prediction accuracy for AGB estimations when combined with Sentinel-2 data [9,10]. Further, SAR datasets with higher penetration, i.e., L-band radar, such as the Advanced Land Observing Satellite (ALOS PALSAR) were successfully applied for AGB estimation in wooded savanna and forest [5,11,12]. Recently, high resolution Spaceborne LiDAR data were provided by the Global Ecosystem Dynamics Investigation (GEDI), allowing 3D modelling of the earth surface with a more precise estimation of the AGB [13,14,15]. Based on its performance, the GEDI level 4A product that includes AGB predictions based on GEDI measurements was released. GEDI Level 4A product could therefore be an alternative to ground truth data for locations where field measurements are not possible, and a powerful dataset for the near-real time monitoring of land use, land-use change, and forestry (LULUCF) at global level.

LULUCF resulting from the conversion of forest into agriculture is acknowledged to contribute to climate change through carbon emissions [14]. For instance, in West Africa 1.71 Gt and 32.5 Kt of CO_2_e was emitted between 2001 and 2021 due to deforestation in Côte d’Ivoire and Burkina Faso, respectively [15]. Forests are converted into agroforestry systems (AFS) for food and cash crop production (cocoa, cashew or mango), and the crops are managed in association with trees [16]. Because of the ubiquity of trees in their composition, AFS are acknowledged to have an interesting potential for carbon sequestration, and are considered as a viable solution for climate change mitigation in West Africa [17,18,19]. They showed higher carbon stocks than crop lands, which could be added to global and national carbon budgets [20,21]. AFS demonstrated their ability to control the temperature by creating a microclimate that is beneficial for other crops [20]. Moreover, AFS are alternative sources of income, improve soil fertility and contribute to the food diet [21].Yet, the methods for estimating AGB in AFS are debated: Nair & Nair [22] argued that the pantropical allometric equations commonly used were not appropriate for AGB modelling, since they were mainly developed for forestry applications. The consequence being the overestimation of AGB in AFS as the allometric equations have been developed using forest trees, which are not found in AFS. The study also pointed out the importance of the stratification based on climatic conditions as a major determinant of AGB density. In fact, the estimation of the AGB at large scale (continental level for example) overlook the influence of climatic regions on AGB levels and thereby introduces uncertainties in the estimation [19]. It was reported that the impact of climatic conditions on the management practices of AFS is not negligeable and needs to be considered for large-scale studies. Moreover, the applicability of RS in AFS is challenging in West Africa due to the spectral similarities among AFS categories. In a recent study, Kanmegne Tamga et al. reported that RS data were unable to effectively separate AFS in West Africa, returning a high level of mixed pixels across the map (more than one AFS in a pixel) [23]. Therefore, RS-based analysis resulted in maps and estimations associated with higher level of uncertainties that should be addressed.

Considering the availability of the new GEDI level 4A product and its potential to improve AGB estimation and monitoring across terrestrial ecosystems, predictions based on this product are required to be assessed and its potential for AGB estimation in AFS of West Africa should be evaluated. Moreover, there is a need for a modelling workflow that captures the spatial distribution of uncertainties in the estimation of AGB. This could improve the ability of parties to assess and report their carbon inventories in line with the GPG. Therefore, the aim of this research is to (i) evaluate the potential of optical, SAR and LiDAR data for AGB estimation in AFS and (ii) estimate the AGB of various AFS in different climatic regions.

## 2. Materials and Methods

### 2.1. Study Area

**Figure 1:**
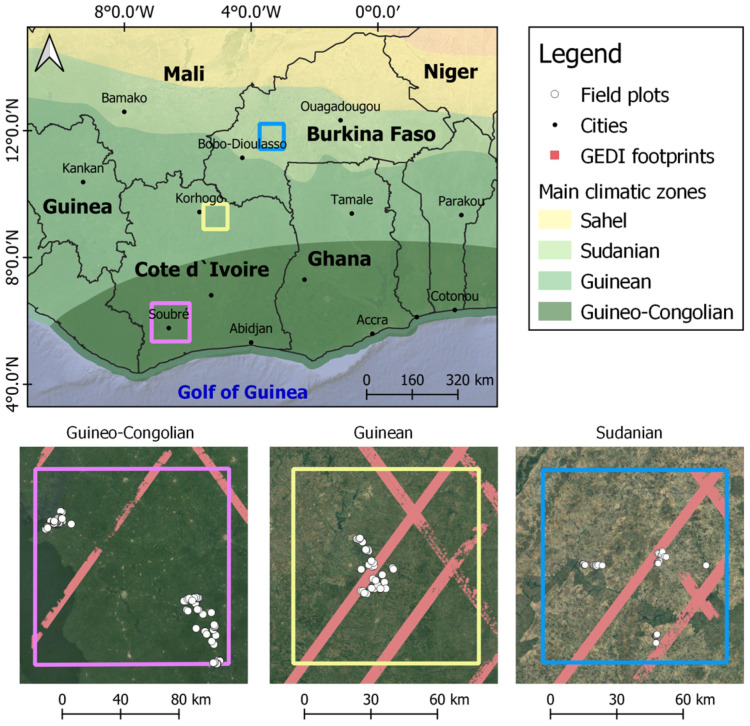
Study area for AGB estimation in West African AFS (Source: Climatic zones in west Africa—Humanitarian Data Exchange).

The study was carried out in Côte d’Ivoire and Burkina Faso, two neighboring countries in West Africa. The study areas comprised three climatic regions: the Guineo-Congolian, the Guinean, and the Sudanian regions following the south-north gradient (Figure 1), since AFS are preferably established under their conditions. The Guineo-Congolian region is the wettest in West Africa, with an annual rainfall between 2200 and 5000 mm. The region has been mostly forested in the past but is currently reduced to small patches. The land has been converted for cash crops production including cocoa, rubber, and oil palm. The food crops represented by cassava, yam, and rice are often grown on small household farms. The Guinean climate is characterized by an annual rainfall between 1200 and 2200 mm. The vegetation is dominated by semi-deciduous forest and wooded savannas with tree heights reaching up to 20 m. The region is severely affected by human activities, particularly slash and burn agriculture, which therefore reduces the extent of forest area. In Côte d’Ivoire, the region is dominated by cashew and mango farming. The Sudanian region is the domain of savanna and is characterized by drier conditions (annual rainfall between 600 to 1200 mm) and a dry season of 5 to 7 months. In Burkina Faso, the landscape is dominated by farms of sesame, cotton, and maize, which come always in association with trees such as shea butter (*Vitellaria paradoxa*), the African locust bean (*Parkia biglobosa*), and the apple-ring acacia (*Faidherbia albida*).

### 2.2. Agroforestry Systems

AFS is a collective name for land-use system, practices, or technologies, where woody perennials (trees, shrubs, bamboo, etc.) are deliberately integrated with agricultural crops and/or animals in the same land management unit in some form of spatial arrangement or temporal sequence [24]. The woody perennials and the associated component (crops and/or animal) are the elements used in the classification of AFS as described by Atangana et al. [25]:

The structural composition and arrangement of the different components in the system, which refers to the tree species and the tree density on the land;

The temporal sequence of introducing the different components;

The function of the woody perennials in the system which could be shade provision, fruit production, firewood, fodder, or soil restoration.

In this study, four AFS agri-silvicultural systems (woody perennials and agricultural crops) were identified in the study areas (Table 1). Home gardens, improved fallow, and multipurpose trees on croplands were referred to as farms, and plantations crop combinations were identified by the main cash crop of the system (cocoa, rubber, oil palm, mango, and cashew).

#### 2.2.1. Plantation Crop Combination 

**Figure 2:**
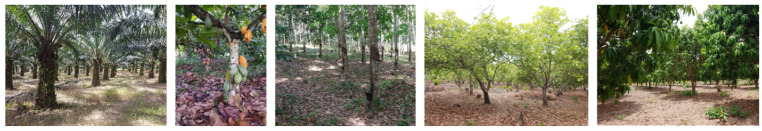
Plantation crop combinations in Côte d’Ivoire. From left to right: oil palm, cocoa, rubber, cashew, and mango plantations. (Pictures from the field campaign, March 2020).

The plantation crops were mainly found in the Côte d’Ivoire. Cocoa, rubber, and oil palm was identified in the Guineo-Congolian region, whereas cashew and mango plantations were mostly located in the Guinean region. They were often installed at the expenses of forest or old fallows. The selected plantations were at least 10 years old, with at least one production cycle (Figure 2). The tree components in the oil palm plantations were managed sequentially in the form of a long rotation that could be up to 25 years.

#### 2.2.2. Farms

**Figure 3:**
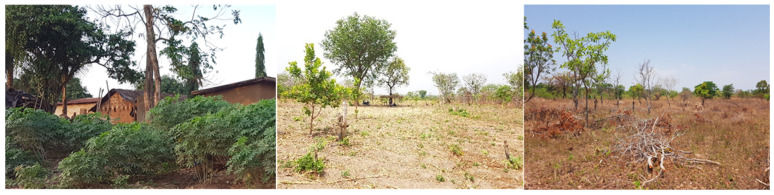
Examples of AFS in West Africa. From left to right: home gardens, multipurpose trees on crop lands, and improved fallow (from where firewood is often collected) (picture from the field campaign, Côte d’Ivoire, March 2020).

The land was mainly managed for food production, including cassava (*Manihot esculenta*), rice (*Oriza sativa*), yam (*Dioscorea alata*), maize (*Zea mays*), and plantain (*Plantago* sp.). In the Sudanian region, some of the farms are exploited for cash crop such as cotton (*Gossypium* sp.) and sesame (*Sesamum indicum*) (Figure 3). Farms were often established on formal crop plantations, when the economic value of the production was no longer interesting for the farmer. Trees were then cut down, and food crops were introduced. Moreover, when the fertility level was not sufficient to sustain the sufficient production level, the farms were left in fallow for a couple of years (up to 7 years) and the land could be converted either into crop plantations or farms.

### 2.3. Data Collection

**Figure 4:**
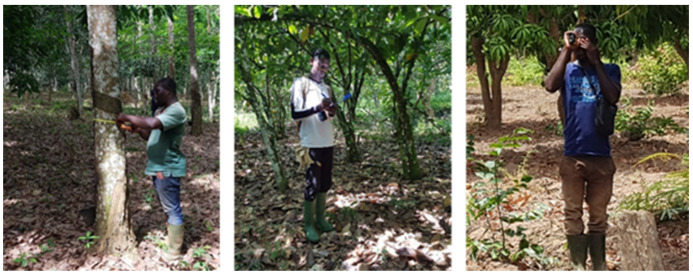
Collection of biophysical parameters of trees in AFS. from left to right: diameter measurement using a diameter tape in a rubber plantation; species identification in a cocoa farm, and height measurement with an altimeter in a mango plantation (field campaign, Côte d’Ivoire, March 2020).

A region of interest was defined for each of the climatic regions, within which two field campaigns were conducted in March 2020 and November 2021. The field campaign consisted of collecting biometric parameters of the trees in different AFS, using sample plots of 40 m × 40 m each. The collected variables (Table A1 in Appendix A) included the diameter at breast height recorded with a diameter tape; the height recorded using an altimeter, and the species name of each tree (Figure 4). 

The specie’s name was used to retrieve the wood density from the ICRAF’S database [26]. For trees that were not found in the database (*Ficus gnaphalocarpa* for example), the wood density of a tree in the same family (*Ficus trachyphylla* in this case) was used. The wood density (σ), the height (H) and the diameter (D) were compiled to determine the aboveground biomass (AGB) using the allometric equation proposed by Aabeyir et al. [27]. The estimated AGB was averaged per sample plot and used for the modelling.

AGB = 0.0580 × σ × (D^2^ × H)^0.999^ (1)

In total, 122 samples were collected in southern Côte d’Ivoire, including 62 farms, 30 cocoa and 30 rubber (Guineo-Congolian region); 66 samples in the northern part of Côte d’Ivoire (21 cashew, 22 mango and 23 farms; Guinean region) and 56 samples in Burkina Faso (Sudanian region), in different crop farms (sesame, cotton and maize) associated with trees.

### 2.4. Satellite Data

Optical data from Sentinel-2 (S2), SAR data from Sentinel-1 (S1), and ALOS PALSAR were considered as independent variables, whereas LiDAR data from the Global Ecosystem Dynamic Investigation (GEDI) was considered as an alternative reference source data (dependent variable). Data from both constellations of the S2 mission (S2A and S2B) were accessed via Google Earth Engine (GEE) to generate a cloud free composite image over the region of interest. The pre-processing of the S2 data was performed based on Sen2cor, which generates a bottom of the atmosphere image corrected atmospheric, terrain, and cirrus corrected, from the top of the atmosphere data. To get the cloud free composite image, the maximum cloud percentage was set at 5%, and all the scenes within the ROI between January to December 2021 were used to generate median images for five bands (blue, green, red, red-edge, and near-infrared). The generated median images were then used to produce seven vegetation indices (NDVI, GLI, VARI, TCARI, MSAVI, EVI, SAVI) at the spatial resolution of 10 m (Table 2). 

The VH/VV and HH/HV polarizations were available for the C-band (S1) and L-band (ALOS) SAR data, respectively, and were resampled at 10 m resolution. The ALOS data were accessed using GEE, where only yearly composites are available and an average image over the past three years (2019 to 2021) was generated to reduce the noise. S1 data were accessed from the Sentinel-1 Global Backscatter Model (S1GBM) [28]. It used the Inferometric Wide swath (IW) as acquisition mode for VH and VV polarizations, which is the main acquisition mode for land monitoring. Composite images for each polarization were generated based on the median pixel value between January and December 2021. The Grey Level Co-occurrence Matrix (GLCM) texture parameters were generated for each of the polarization as a mean to increase the feature space of S1 and ALOS (Table 3) for the AGB estimation. The parameters were computed using the GLCM R package version 1.6.5 using a window size of 5 by 5 pixels and the default number of grey levels of 32, where all four directions and an offset of 1 were considered [29].

The GEDI data were downloaded from Earth Data platform (https://daac.ornl.gov/cgi-bin/dsviewer.pl?ds_id=2056, last accessed on 19 December 2022), and the shapefile of the footprints was extracted. The LiDAR system of GEDI is made of three lasers that produce eight parallel tracks of observations. Each laser is emitted at a frequency of 242 Hz to illuminate a 25 m footprint where the 3D structure is measured. Each footprint is separate by 60 m along the track and 600 m across-track distance. From the measured 3D structure, predictions of the aboveground biomass density (Mg ha^−1^) and the prediction standard error were generated for each footprint. The footprint’s AGB were derived from parametric models that related simulated GEDI level 2A waveform relative height (RH) metrics to field plot estimation of AGB [31]. 

### 2.5. Overview of the Methodology

The analysis approach assessed the reference AGB estimations from two different sources: (i) field measurements and (ii) level 4A product (GEDI L4A). For each reference source, Optical and SAR data were used as predictors of AGB to train different machine learning (ML) regression algorithms for AGB estimations in the region of interest. The AGB reference from the field data was used to assess the AGB predictions extracted from the GEDI L4A product. Finally, AGB and RMSE maps were generated.

### 2.6. Data Processing

Spaceborne optical data (bands and vegetation indices) and SAR data (intensity and GLCM textures) were evaluated both individually and as combinations (ALOS + S2 and S1 + S2). The shapefile of the field polygons was used for pixel value extraction when the field data were used as AGB reference, and the GEDI footprints were used for the scenario with GEDI L4A as AGB reference. The extracted values were used for tuning the ML regression models by using 75% of the data for training and 25% for testing. 

Three types of ML regression models were trained: (i) A linear regression model for which three regularization techniques were explored: Ridge, Lasso, and ElasticNet [32]. The model training started with a stepwise regression to remove redundant variables. The relevant variables were then used to train the models. (ii) A random forest (RF) algorithm was trained using a repeated cross validation method of 10 folds and 10 repetitions. Using the root mean square error (RMSE, Formula (2)) as an evaluation metric, the optimal number of features per node was identified. A variable selection using RF (VSURF) was also implemented. The approach aimed at eliminating redundant variables from the dataset by selecting the variables that were mostly related to the response. Then, this selection was refined by reducing redundancy in the selected variable for the prediction [33]. (iii) A support vector machine classifier (SVM) was applied, which aimed at minimizing the margin between two classes to distinguish them [34].

The prediction performance of each model was evaluated based on their R^2^ (Formula (3)) and RMSE. The model with the best score (higher R^2^ and lower RMSE) was used to generate the AGB map of the study area. In addition, the spatial distribution of the prediction uncertainties was modelled [23]. For that, the field data reference AGB estimation was used to assess the AGB prediction, and the difference was used to generate the RMSE for each plot. The RMSE of each plot was then used as reference to model the RMSE on the map.

(2) $$\mathrm{RMSE}=\sqrt{\frac{{\sum \;\left(\hat{y}p-\mathrm{yp}\right)}^{2}}{n}}$$

(3) $$R^{2}=1-\frac{\sum {\left(\hat{y}p-\mathrm{yp}\right)}^{2}}{\sum {\left(\mathrm{yp}-\bar{y}p\right)}^{2}}$$

where $\hat{y}$p is the predicted AGB value at the pixel ip; yp is the field AGB estimation value, n is the number of pixels in the field sample, and $\bar{y}$ = the average value of AGB in the field plot.

## 3. Results

### 3.1. A global Model for AGB Estimation in West African AFS

**Figure 5:**
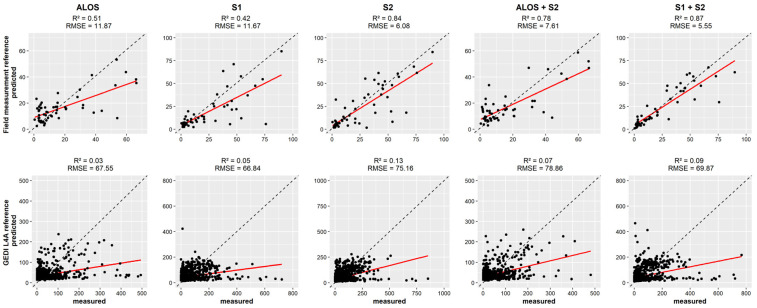
Scatterplot showing the relation between the measured and predicted AGB in AFS in West Africa for optical and SAR data. The first row uses field measurements as AGB reference, and the second row uses the GEDI L4A product.

The prediction performance of AGB using a global model for the entire West African AFS was evaluated by plotting measured vs. predicted values for each of the AGB reference sources (Figure 5). AGB estimations generated using the field measurements as AGB reference showed the highest scores for the combination S1 + S2 (R^2^ = 0.87 and RMSE = 5.55), while S2 (R^2^ = 0.84, RMSE = 6.08) showed higher scores than ALOS+S2 (R^2^ = 0.78, RMSE = 7.61). Considering the SAR data, ALOS (R^2^ = 0.51, RMSE = 11.87) showed a higher score than S1 (R^2^ = 0.41, RMSE = 11.67). When using GEDI L4A product as AGB reference, a weak relationship was observed between the optical and SAR data and the GEDI L4A AGB predictions. The best score that was achieved was with S2 data (R^2^ = 0.13, RMSE = 75.16). Other datasets returned accuracies below 10%, and the error level was found to be 6 to 12 times higher than the estimation based on field measurements (for ALOS and S1 + S2 respectively). 

### 3.2. AGB Modelling within Each Climatic Region 

**Figure 6:**
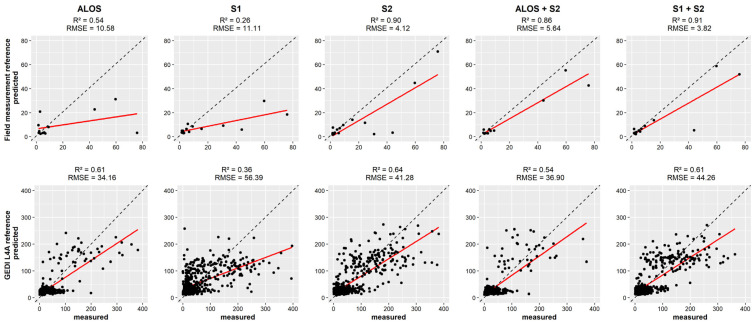
Scatterplot showing the relation between the measured and predicted AGB values in AFS in the Guineo-Congolian region. Dashed line represents the 1:1 line.

The study area was stratified by climatic regions, in which AGB models were separately developed. In the Guineo-Congolian region, the combination S1 + S2 using field measurement as AGB reference showed the highest score (R^2^ = 0.91, RMSE = 3.82). In addition, S2 (R^2^ = 0.90, RMSE = 4.12) showed higher performance than the combined ALOS+S2 (R^2^ = 0.86, RMSE = 5.64). It was found that few observations were used to assess the AGB predictions with field measurements as reference (Figure 6). When using GEDI L4A as reference, S2 (R^2^ = 0.64, RMSE = 41.28) showed the highest score. ALOS (R^2^ = 0.61, RMSE = 34.16) and S1 + S2 (R^2^ = 0.61, RMSE = 44.26) showed similar accuracies, yet ALOS model showed a smaller error. 

**Figure 7:**
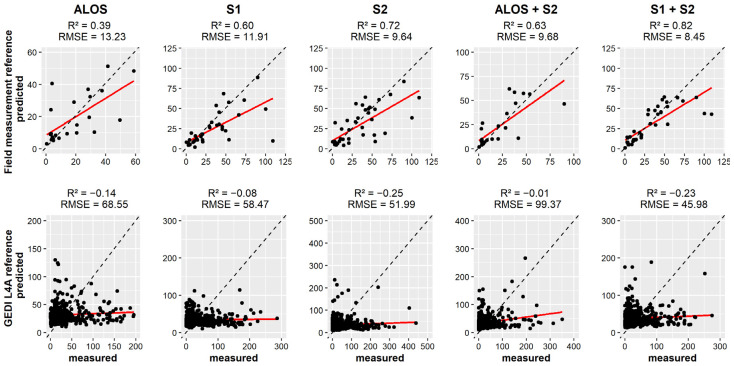
Scatterplot showing the relation between the measured and predicted AGB values in the Guinean region.

In the Guinean region, the combination S1 + S2 (R^2^ = 0.82, RMSE = 8.45) showed the highest score, followed by S2 (R^2^ = 0.72, RMSE = 9.64) and ALOS+S2 (R^2^ = 0.63, RMSE = 9.66) for AGB estimations based on field measurements (Figure 7). The model slightly overestimated AGB of plots with an AGB lower than 25 Mg ha^−1^. When using GEDI L4A predictions as reference, all the considered datasets gave negative accuracies suggesting that the model’s predictions are worse than guessing, which is expressed by larger RMSE values. 

**Figure 8:**
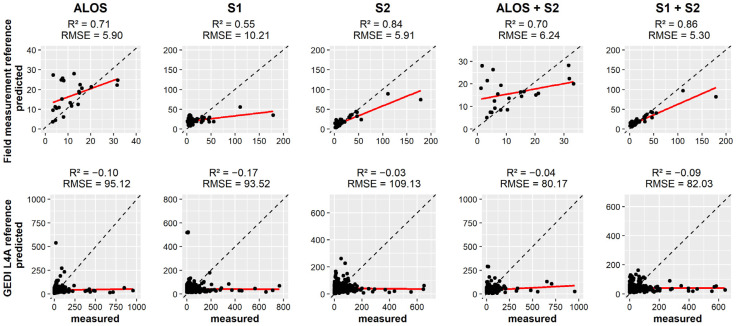
Scatterplot showing the relation between the measured and predicted AGB values in the Sudanian region.

In the Sudanian region (Figure 8), The highest score was associated with S1 + S2 (R^2^ = 0.86, RMSE = 5.30), followed by S2 (R^2^ = 0.84, RMSE = 5.91). ALOS and ALOS+S2 overestimated AGB for plots with an AGB values lower than 15 Mg·ha^−1^. With GEDI L4A predictions as AGB reference, the model predictions were worse than guessing like the Guinean region. 

### 3.3. Performance of the Algorithms

**Figure 9:**
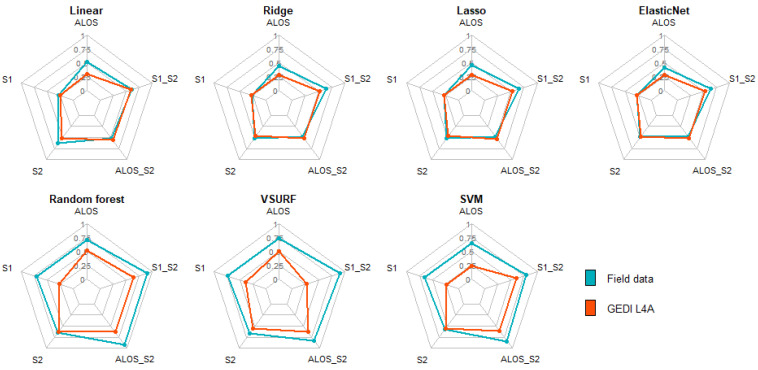
R^2^ from ML algorithms on different datasets for AGB reference sources (Field data and GEDI): ALOS_S2: combining ALOS and S2; S1_S2: combining S1 and S2.

Figure 9 summarizes the performance of ML algorithms for AGB modelling in AFS. Higher R^2^ score was reached when optical and SAR data were combined. With linear-based models, the accuracies were comparable for the two AGB reference sources. The combination of optical and SAR (ALOS + S2 and S1 + S2) data showed the highest score for random forest when the prediction was based on field measurements (R^2^ = 0.91). When using GEDI L4A as reference, the accuracies were similar for S2, ALOS+S2 and S1 + S2 (R^2^ = 0.63).

**Figure 10:**
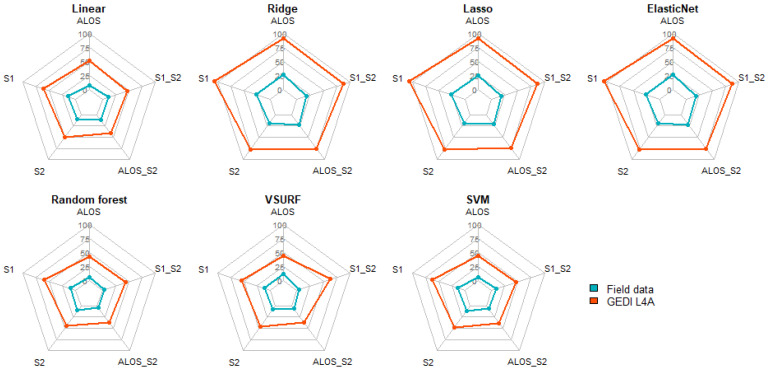
RMSE from ML algorithms on different dataset for AGB reference sources (field data and GEDI).

RF showed the smallest prediction error across both AGB reference source (Figure 10). The smallest RMSE value was obtained when using field data and S1 + S2 (RMSE = 3.78), while the same combination returned ca. ten times higher errors (RMSE = 37.28) when evaluating against GEDI L4A data.

### 3.4. AGB Maps and Uncertainties

**Figure 11:**
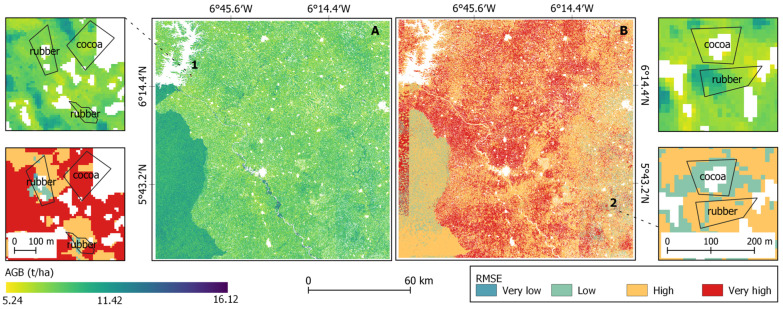
AGB map in the Guineo-Congolian region (South Côte d’Ivoire) (**A**) with the uncertainty map (**B**).

The AGB map in the Guineo-Congolian region was generated (Figure 11A) with the corresponding uncertainty map (Figure 11B), where the RMSE value of the model (3.82) was used as threshold to classify the error [23]. The error level was high above the threshold, while the error level below the threshold was low. The analysis suggested that cocoa and rubber farms have a relatively high prediction error. 11.29% of the cocoa samples and 16.67% of the rubber samples showed a low RMSE, compared to 48.39% for agricultural farm. The snippet 1B showed a very high prediction error occurred mainly in cocoa and rubber plots. 

**Figure 12:**
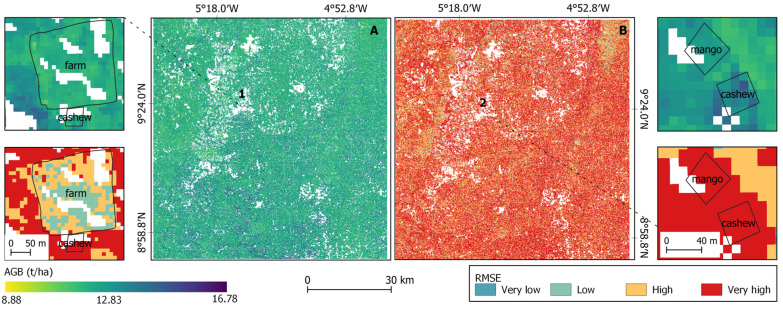
AGB map in the Guinean region (north Côte d’Ivoire) (**A**) with the uncertainty map (**B**).

In the Guinean region, the map showed that the AGB level was higher with a minimal value of 8.88 Mg ha^−1^ (Figure 12). The prediction error was found to be very high in cashew and mango plantations (snippet 1A), whereas the error in the agricultural farm was low (snippet 1B).

**Figure 13:**
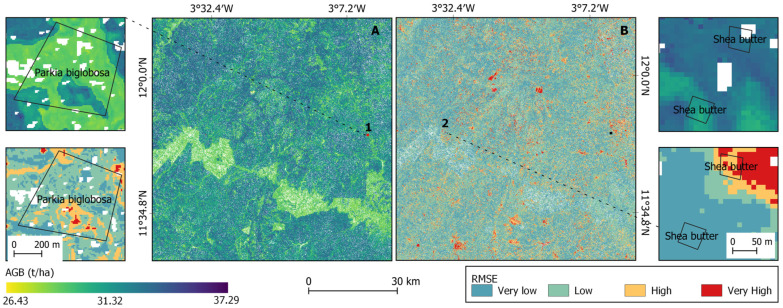
AGB map in the Sudanian region (west Burkina Faso) (**A**) with the uncertainty map (**B**).

In the Sudanian region, the uncertainty map revealed that the prediction error was very low over the study area (Figure 13). It was found that shea butter (*Vitellaria paradoxa*), the AFS dominant species in the region, showed some plots with smaller prediction error and other with high error for AGB estimation (snippet 2B). The image also showed that the uncertainty level varied within AFS. Snippet 1A showed that an AFS based on *Parkia biglobosa* showed a high spatial heterogeneity of the uncertainty level.

### 3.5. AGB Estimations in Different Agroforestry Systems

The AGB in different AFS of West Africa and the corresponding R^2^ and RMSE is presented in Table 4. In the Guineo-Congolian climate region, cocoa plantations (7.51 ± 0.6 Mg ha^−1^) showed a higher AGB compared to rubber (7.33 ± 0.33 Mg ha^−1^) and agricultural farms (6.97 ± 0.42 Mg ha^−1^). However, the prediction performances were higher in agricultural farms (R^2^ = 0.76, RMSE = 7) and cocoa (R^2^ = 0.6, RMSE = 7.48) compared with low performance in rubber plantations (R^2^ = 0.25, RMSE = 13.86).

In the Guinean region, the highest AGB was found in cashew plantations (13.78 ± 0.98 Mg ha^−1^), mango plantations (12.82 ± 0.65 Mg ha^−1^), and agricultural farms (11.78 ± 0.19 Mg ha^−1^). Agricultural farms in this region showed higher AGB values compared to the Guineo-Congolian region, with a difference of about 4.81 Mg ha^−1^. A higher AGB prediction accuracy was recorded in farm (R^2^ = 0.78, RMSE = 6.62) and mango (R^2^ = 0.58, RMSE = 21.07), while the estimation in cashew plantations (R^2^ = 0.37, RMSE = 38.68) showed larger errors.

The AFS in the Sudanian region were differentiated based on the dominant tree in the agricultural farm. In fact, the difference in the AGB density was large depending on the tree species managed on the farm. As an example, a difference of 75 Mg ha^−1^ was found between AFS based on African custard-apple (*Annona senegalensis*) and farms with Marula (*Sclerocary birrea*). Shea butter (*Vitellaria paradoxa*) is the most popular tree managed in AFS in the region and showed an AGB density of 15.05 ± 7.34 Mg ha^−1^. The average AGB density in the region was around 34.19 Mg ha^−1^, which was ca. 148% higher than cashew plantations, 167% higher than mango plantations, 355% higher than cocoa plantations, and 366% higher than rubber plantations.

**Figure 14:**
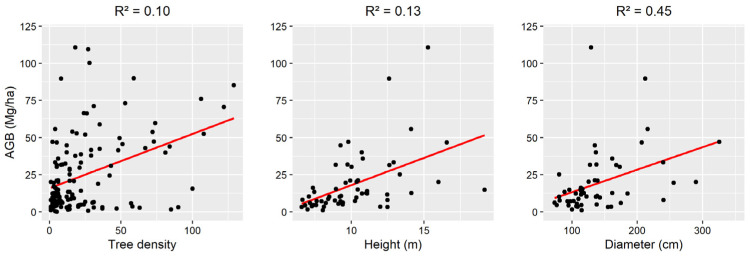
Relation between tree parameters and AGB in the AFS of West Africa.

The relation between the biometric parameters of the trees (height and diameter) and the composition of the different AFS (tree density) was investigated (Figure 14). It was found that the diameter of the trees was the major determinant of AGB in AFS of West Africa (R^2^ = 0.45). The height and the tree density showed a comparable coefficient of determination that was R^2^ = 0.13 and R^2^ = 0.1 respectively. 

## 4. Discussion

This study pointed out the importance of stratification of the region of interest for AGB modelling across AFS in West Africa. Large-scale studies (at regional or national levels) tend to overlook the effect of climatic conditions, which is particularly detrimental for AGB modelling in AFS. Balima et al. [35] found that climatic conditions explained about 3% of the variation in carbon stocks. For larger regions of interest, the variation of the climatic conditions within the region of interest should be minimized [36]. Because of stratification, the prediction error was reduced by 31.17% in the Guineo-Congolian region. It was also found out that AGB was better explained by S2 than S1 and ALOS data. This was in line with Forkuor et al. [36], who reported that S2 was a better predictor of AGB than S1 in the Sudanian region. This finding is supported by the available literature where S2-derived indices were reported to better explain the variance in AGB compared to S1, both in AFS and forest [9,10]. Moreover, the combination of optical and SAR data performed better than single satellite products, as supported by the recent relevant literature [5,10]. Concerning the ML algorithms, RF was the most efficient algorithm for AGB estimation. VSURF showed the same accuracy as RF, but with a larger error, possibly because of the dimension reduction during its implementation. Linear-based algorithms (Linear, Ridge, Lasso, and ElasticNet) were unable to model the AGB, especially when the GEDI L4A predictions were used as reference data. Those algorithms fail to model non-linear relationships, since they use a piecewise linear solution to generate the predictions [32,36].

The AGB estimations by GEDI L4A product underperformed when used as reference compared to field measurements. An accuracy of 13% was obtained with S2 data. Its performances in the Guinean and Sudanian regions resulted in a negative R^2^, showing that the model prediction was very large compared to the measured values. However, the Guineo-Congolian region, a coefficient of determination around 64% was reached using S2 data. By using the GEDI L4A product as AGB reference source, the prediction error was found to be 8.94 times higher than when using field data. This could result from the fact that biomass prediction in GEDI L4A product is derived from tree canopy height, so that ecosystems with a low tree density showed a small coefficient of determination (R^2^) and a large error, while the AGB predictions in denser forests and wooded savannas would be more accurate [37,38]. Concerning the AGB estimation based on field measurements, a locally developed allometric equation [27] was used to calculate AGB using the biometric variables collected during field campaigns. The mixed-species allometric model developed by [27] in Ghana was tested and shown to be equivalent to the pantropic model of Chave et al. [39], while showing higher mean values for the predictions. Furthermore, the latter study was carried out in the Guinean climatic region, and the tree species used to develop the allometric model were found in the AFS across West Africa. 

Concerning the AGB in different AFS of West Africa, cocoa plantations were shown to have an AGB level 12 times lower than the average AGB in cocoa agroforestry [39,40]. This is explained by the fact that cocoa trees in Côte d’Ivoire are grown in pure stand (full sun cocoa) where all other trees are removed, which is not the case in typical cocoa AFS. The AFS in the Sudanian region showed a lower tree density (35 trees ha^−1^) compared to cocoa plantations (368 trees ha^−1^), while their average AGB was 4.7 times higher (34.19 Mg ha^−1^) than cocoa plantations (7.51 Mg ha^−1^). This was explained by the fact that the size of the trees diameter (45%) and height (13%) in AFS was more related to the AGB than tree density (10%). Cocoa trees are often small (diameter < 50 cm and height ≈ 1.7 m), while the average tree is larger for the AFS in the Sudanian region (diameter ≈ 1.4 m and a height ≈ 10.19 m). These findings are in line with Balima et al. [35] who found that diameter and height describe to 69.52% and 27.77% of carbon variation respectively in woody ecosystems in Burkina Faso. This also reveals a potential increase of the AGB in cocoa plantations in Côte d’Ivoire by introducing and maintaining forest tree species such as white silk-cotton tree (*Ceiba pentandra*) or kola (*Cola accuminata*) [41]. 

In addition, we found very low accuracies of the AGB predictions for rubber, cocoa, cashew, and mango plantations at the sample plot level. The highest prediction error was found in the Guinean region across cashew and mango plantations. The presumable reason is that the AFS classes were under-sampled, which lead to an increase in the prediction errors [42]. For complex landscapes such as AFS, the spectral signatures are quite similar, which make it challenging to separate one AFS from the other, thus creating confusions between classes [23]. Even if increasing the sample size will reduce the sample error, a spatial assessment of the prediction error is crucial to generate a meaningful and applicable AGB map in AFS.

## 5. Conclusions

The aim of the study was to develop and apply a workflow for the AGB estimation in the AFS of West Africa. Two crucial aspects were investigated: (i) Evaluating the potential of optical, SAR and LiDAR data for AGB estimation in AFS; and (ii) estimating the AGB of different AFS in diverse climatic regions. The analysis revealed that the stratification of the study area based on the climatic regions reduced the prediction error up to ca. 31.17% in the Guineo-Congolian region. The AGB predictions from GEDI L4A product used as reference underperformed across the different AFS in West Africa. A performance of 64% was achieved in the Guineo-Congolian region, but the prediction error level was 8.94 times higher compared to the estimation based on field measurements. In addition, the combination of S1 and S2 data returned the best score for AGB estimation in the different AFS in West Africa. The comparison of the algorithm showed that linear-based ML algorithms such as Ridge, Lasso, and ElasticNet were unable to model AGB in AFS, while RF returned the highest R^2^ and the smallest RMSE. The study also revealed that the diameter was the most important determinant of AGB compared to height and tree density. This was the reason why the AFS in the Sudanian region showed higher AGB values than cocoa, cashew, and mango plantations. Moreover, the error level in the AGB predictions could be attributed to under-sampling, but the main reason was the difficulty to differentiate AFS classes with the applied RS-based analysis due to the similarities within their spectral signatures. Therefore, an uncertainty map should be provided alongside the AGB map to provide information about the spatial distribution of the prediction error across the study area. Conclusively, the study pointed out the under-exploited potential of improving the AGB in AFS by introducing and maintaining forest tree species in cocoa plantations in Côte d’Ivoire as the world’s largest producer. For modelling and monitoring of the AGB in AFS in West Africa, the stratification of the region based on the climatic conditions is recommended, and the combination S1 + S2 data with a RF model is proposed. This study improved the good practice guidance for AGB estimations by providing a framework for RS-based AGB estimation in AFS.

## Figures and Tables

**Table 1:** AFS identified in West Africa and their description [25].

AFS	Description
1. Home gardens	Combination of trees and crops around farmer’s house. The woody components are often fruit trees.
2. Improved fallow	Perennial planted or left to grow during fallow. The woody components are fast-growing leguminous tree species
3. Multipurpose trees on croplands	Trees scattered in cropland. The perennial components are multipurpose trees (fruits, medicine, fodder, firewood, etc.)
4. Plantation crop combinations	Mixture of trees and cash crop such as cocoa, rubber, mango, and cashew. The associated tree species are often forest tree species.

**Table 2:** Vegetation indices and formulae (bands B: blue, G: green, R: red, RE: red-edge, NIR: near infra-red).

Vegetation Indices	Formula
1. Normalized Difference Vegetation Index (NDVI)	(NIR − R)/(NIR + R)
2. Green Leaf Index (GLI)	(2 × G − R − B)/(2 × G + R + B)
3. Enhanced Vegetation Index (EVI)	2.5 × (NIR − R)/(NIR + 6 × R − 7.5 × B + 1)
4. Soil Adjusted Vegetation index (SAVI)	(1 + L) × (NIR − R)/(NIR + R + 0.5)
5. Modified Soil Adjusted Vegetation Index (MSAVI)	0.5 × (2 × NIR + 1 − sqrt ((2 × NIR + 1)^2^ − 8 × (NIR − R)))
6. Transformed Chlorophyll Absorption in Reflectance Index (TCARI)	3 × ((RE − R) − 0.2 × (RE − G) × (RE/R))
7. Visible Atmospherically Resistance Index (VARI)	(G − R)/(G + R − B)

**Table 3:** GLCM texture parameters [30] where (i): reference cell in the co-occurrence matrix; (j): neighbor cell in the co-occurrence matrix; Pij: GLCM expressed as a probability of having a pair of cell values with a specific value in a specific spatial relationship, µ: mean and σ: standard deviation within GLCM in the specified window size.

Texture Measures	Formula	Explanation
Entropy	∑ − ln(P_ij_) P_ij_	Smaller P_ij_ leads to higher entropy value
Contrast	∑P_ij_ (i − j)^2^	Express difference as an exponential function
Variance	∑P_ij_ (i − µ_i_)^2^	Describe the variance of GLCM values
Correlation	∑P_ij_ [(i − µ_i_) (j − µ_i_)/σ^2^]	Describe the correlation of GLCM values
Mean	∑P_ij_/N	Describe the mean of the GLCM values
Homogeneity	∑P_ij_/(1 + (i − j))^2^	Express difference as an inversed exponential function
Dissymmetry	∑(∑(|i − j| P_ij_))	Express difference as a linear function
Second moment	∑(∑(P_ij_)^2^)	Return the max value when all pixels are identical

**Table 4:** Summary of AGB estimations for the field plots investigated in different AFS of West Africa.

Climatic Region	AFS	Carbon (Mg ha^−1^)	R^2^	RMSE	N Plots
Guineo-Congolian	Farm	6.97 ± 0.42	0.76	7.00	62
	Cocoa	7.51 ± 0.6	0.6	7.48	30
	Rubber	7.33 ± 0.33	0.25	13.86	30
Guinean	Cashew	13.78 ± 0.98	0.37	38.68	21
	Mango	12.82 ± 0.65	0.58	21.07	22
	Farm	11.78 ± 0.19	0.78	6.62	23
Sudanian	Custard apple	82.11			1
	Shea butter	15.05 ± 7.34			11
	Apple-ring	23.24 ± 10.3			5
	Marula	6.59 ± 0.34			2
	African locust bean	43.97 ± 54.38			6

## Data Availability

Not applicable.

## Appendix A

**Table A1:** Data sheet of the field campaign.

GENERAL INFORMATION					
Plot ID		date (dd-mm-yy)		Country	
				village	
Area (ha)		Picture N°		Coord W	
				Coord N	
TREE SPECIES					
Type of agroforestry system					
N° of trees					
N° of species					
Function of trees					
IF FARM PLOT, FILL HERE					
Current crops					
Previous crops					
Associated crops					
rotation					
AFS MANAGEMENT					
Age of the parcel (in years)					
length of cultivation					
Treatments (manure, fertilizer, pesticides, …)		Manure: Fertiliser: Pesticides:			
Quantity of fertilizer and pesticides		Pesticides: kg Fertiliser:			
INFORMATION ON TREES					
ID	Local/ common name	GPS coordinates	Picture N°	Diameter (m)	Height (m)
1					
2					
